# Quantitative melanoma diagnosis using spectral phasor analysis of hyperspectral imaging from label-free slices

**DOI:** 10.3389/fonc.2023.1296826

**Published:** 2023-11-17

**Authors:** Bruno Schuty, Sofía Martínez, Analía Guerra, Federico Lecumberry, Julio Magliano, Leonel Malacrida

**Affiliations:** ^1^ Unidad de Bioimagenología Avanzada, Institut Pasteur de Montevideo, Hospital de Clínicas Universidad de la República, Montevideo, Uruguay; ^2^ Unidad Academica de Dermatología, Hospital de Clínicas, Facultad de Medicina, Universidad de la República, Montevideo, Uruguay; ^3^ Instituto de Ingeniería Eléctrica, Facultad de Ingeniería, Universidad de la República, Montevideo, Uruguay; ^4^ Unidad Academica de Fisiopatología, Hospital de Clínicas, Facultad de Medicina, Universidad de la República, Montevideo, Uruguay

**Keywords:** skin cancer, melanoma, fluorescence microscopy, hyperspectral imaging, phasor analysis, spectral phasor, nevus, cancer

## Abstract

**Introduction:**

Melanoma diagnosis traditionally relies on microscopic examination of hematoxylin and eosin (H&E) slides by dermatopathologists to search for specific architectural and cytological features. Unfortunately, no single molecular marker exists to reliably differentiate melanoma from benign lesions such as nevi. This study explored the potential of autofluorescent molecules within tissues to provide molecular fingerprints indicative of degenerated melanocytes in melanoma.

**Methods:**

Using hyperspectral imaging (HSI) and spectral phasor analysis, we investigated autofluorescence patterns in melanoma compared to intradermal nevi. Using UV excitation and a commercial spectral confocal microscope, we acquired label-free HSI data from the whole-slice samples.

**Results:**

Our findings revealed distinct spectral phasor distributions between melanoma and intradermal nevi, with melanoma displaying a broader phasor phase distribution, signifying a more heterogeneous autofluorescence pattern. Notably, longer wavelengths associated with larger phases correlated with regions identified as melanoma by expert dermatopathologists using H&E staining. Quantitative analysis of phase and modulation histograms within the phasor clusters of five melanomas (with Breslow thicknesses ranging from 0.5 mm to 6 mm) and five intradermal nevi consistently highlighted differences between the two groups. We further demonstrated the potential for the discrimination of several melanocytic lesions using center-of-mass comparisons of phase and modulation variables. Remarkably, modulation versus phase center of mass comparisons revealed strong statistical significance among the groups. Additionally, we identified the molecular endogenous markers responsible for tissue autofluorescence, including collagen, elastin, NADH, FAD, and melanin. In melanoma, autofluorescence is characterized by a higher phase contribution, indicating an increase in FAD and melanin in melanocyte nests. In contrast, NADH, elastin, and collagen dominate the autofluorescence of the nevus.

**Discussion:**

This work underscores the potential of autofluorescence and HSI-phasor analysis as valuable tools for quantifying tissue molecular fingerprints, thereby supporting more effective and quantitative melanoma diagnosis.

## Introduction

In recent decades, there has been a remarkable rise in the global incidence of melanoma skin cancer (1–5). This alarming trend has resulted in an elevated risk of one in 50 individuals in various Western societies. Melanoma, classified as a malignant melanocytic proliferation, can originate from melanocytes in the skin, mucosal tissues, or nervous system (3). Cutaneous melanoma is the most common form of melanoma and is characterized by aggressive behavior, propensity for metastasis, and significant impact on the patient’s overall prognosis, particularly when early detection proves elusive (6–9).

When suspicious skin lesions are detected, standard clinical practice involves removing the lesion with a small margin for histological studies to confirm or rule out melanoma (10, 11). Although clinical evaluation is the initial diagnostic approach for cutaneous melanoma, the gold standard for diagnosis has consistently relied on histological examination under a microscope, depending on the architectural and cytological criteria (12). This process demands the expertise of a skilled and dependable professional and involves a notable waiting period. In cases of suspicion, additional immunohistochemical staining is typically performed (13). Nevertheless, dermatologists currently lack specific markers that would enable reliable differentiation between benign common nevi and malignant melanocytic lesions, such as melanomas. Consequently, false-positive and undetected malignant lesions showed notable prevalence (12, 14). The new diagnostic criteria for melanocytic lesions introduce a distinct category for lesions that defy classification, aiming to address this challenge (15). Far from this being a solution, there is an urgent need for measurable and objective criteria to assist dermatologists in the melanoma diagnostic (12, 16, 17). Hence, we propose the use of hyperspectral imaging and phasor plot analysis as valuable tools to address this problem. This approach enables the evaluation of structural characteristics and allows for the identification of biochemical fingerprints, all in a label-free manner, through tissue autofluorescence fingerprinting (18).

Autofluorescence offers a non-invasive means to monitor metabolic shifts within cells, avoiding the necessity of applying extrinsic fluorophores (19–23). Flavin adenine dinucleotide in its oxidized form (FAD+) and nicotinamide adenine dinucleotide in its reduced form (NADH) are natural fluorophores present in human cells (24). The combination of NADH and FAD+ fluorescence intensity and lifetime describes the shifts in cellular energy metabolism between oxidative phosphorylation and glycolysis (25–27). The application of NADH, being well studied for fluorescence lifetime imaging microscopy (FLIM), combined with FAD+ as an endogenous marker, opens the possibility of distinguishing cell populations such as in cancer and neurodegenerative diseases (24, 28–30). The most abundant studies have been performed on live cell imaging with time-resolved fluorescence (lifetime measurement), including one study by Seidenari et al. conducted on melanocytic lesions (31). While the translation of the metabolic significance of autofluorescence from *in vivo* to fixed samples is currently limited (32, 33), its potential for the development of novel label-free imaging techniques as molecular fingerprints for medical applications, particularly in the field of oncology, holds significant promise (23).

Studies focusing on the skin have revealed the presence of additional autofluorescent molecules including melanin and elastin (34–36). Various investigations have examined their presence within melanocytic lesions using diverse imaging techniques, primarily morphological assessments (37). The main endogenous fluorosphore of melanocytes is melanin, a pigment synthesized by melanocytes inside special intracellular structures called melanosomes. This pigment has different forms, mainly eumelanin and pheomelanin (38). Melanin synthesis favors pheomelanin in malignant melanocytic lesions. This shift has been associated with increased fluorescence emission in melanomas in the red spectrum, and has been explored by other authors as a potential indicator of malignancy *in vivo* (39–41). However, studies investigating the autofluorescent properties in fixed tissues have not yet been conducted in melanocytic lesions.

Hyperspectral imaging (HSI) is a powerful tool for studying autofluorescence because of its unique ability to capture the spectrum of single fluorophores (42–44). In contrast to more complex techniques, such as FLIM, it is simple to use and available in almost all commercial microscopes in the market (45). Nonetheless, data analysis from autofluorescence could be challenging and knowledge-demanding when proposing a proper unmixing approach (43). Spectral phasor analysis for HSI is a novel tool that can be used to study tissue autofluorescence owing to its model-free approach (23, 46). Phasor analysis allows spectrum transformation to a complex number, the G and S coordinates (x and y, respectively) (45, 47). This transformation enables the differentiation of autofluorescent molecules according to their emission spectrum characteristics, namely, the center of mass and bandwidth (45). Owing to its vector properties, it is possible to identify specific phasor fingerprints in the phasor plot for each autofluorescent molecule, and linear combination rules enable the identification and quantification of the component fraction in a simple manner (23, 45). Moreover, the principle of reciprocity can be used to generate images of skin tissues regarding the molecular fingerprints identified in the phasor plot (48). All these properties point to HSI-phasor analysis as an excellent tool to provide new parameters that can support dermatologists in identifying molecular markers for melanoma diagnosis.

Our study aimed to recognize autofluorescence fingerprints using HSI and phasor analysis in melanocytic lesions that can enable nevi and melanoma differentiation. First, we developed an HSI acquisition pipeline that enables label-free whole-slide imaging using a commercial microscope. Using phasor analysis of HSI data, we compared melanoma and nevus molecular fingerprints and identified the key features at the phasor to quantify the differences. Using skin molecular markers, we confirmed the identity of the autofluorescent fingerprints shown in the nevi and melanomas in the phasor plot. Finally, we performed a statistical analysis to compare the numbers obtained by the molecular signatures in the phasor plot (modulation and phase), underscoring the significance and potential of our new approach.

## Materials and methods

### Dataset collection

#### Skin tissue sample

Samples were provided by the Unidad Academica de Dermatología at the Hospital de Clínicas “Dr. Manuel Quintela” in Montevideo-Uruguay. Benign and malignant melanocytic lesions of patients of any age, females and males, biopsied between 2015 and 2018, were selected. Since nevi biopsies were considerably more frequent, we selected 10 nevi per year. Either junctional, compound, or intradermal nevi were included in cases of benign lesions. The exclusion criteria were the presence of dysplasia in the case of nevi and extensive regression (>25%) *in situ* melanomas, melanomas arising within a nevus, and amelanotic melanomas. A number was assigned for each lesion from each dataset, and five benign lesions and five malignant lesions were randomly selected using an online randomizer selector. Five melanomas were included, two of which were ulcerated with a mean Breslow thickness of 1.4 ± 2.22. Five nevi were included and their histological subtypes are listed in **Table 1**. In all cases, histological diagnosis was established by an experienced dermatopathologist, members of the Unidad Academica de Dermatología at the Hospital de Clínicas “Dr. Manuel Quintela,” and then supervised by one of the Assistant Professors.

**Table 1:** Number of skin samples included and their histological diagnosis.

Melanocytic Nevus		Invasive Melanoma	
Lesion	Histological subtype	Lesion	Breslow Thickness
*IN1*	Intradermal	*IM1*	0.9 mm
*IN2*	Intradermal	*IM2*	1.4 mm
*IN3*	Intradermal	*IM3*	6.0 mm
*IN4*	Intradermal	*IM4*	2.5 mm
*IN5*	Intradermal	*IM5*	0.5 mm

Histological subtype is included for melanocytic nevi, and Breslow thickness is included for melanomas. IN, intradermal nevus; IM, for invasive melanoma.

#### Skin biomolecular components

The molecular pure components used for hyperspectral fingerprint identification were collagen from human placenta Type IV (C7521), collagen from human placenta Type III (C4407), collagen from chicken sternal cartilage Type II (C9301), collagen from rat tail Type I (C7661), elastin (E7402) from human skin, and synthetic melanin (M8631). All samples were acquired from Sigma-Aldrich. β-Nicotinamide adenine dinucleotide (NADH) disodium salt, approximately 100% (N8129); and grade I, Roche Diagnostics. Flavin adenine dinucleotide (FAD+) disodium salt hydrate 95% (F6625), from Sigma Aldrich.

### Samples preparation

#### Skin biomolecular components preparations

Approximately 1 mM of NADH or FAD was prepared in phosphate buffer (PBS), 100 mM pH 7.4. Melanin is difficult to prepare in several solvents; therefore, powdered melanin was prepared in 1N ammonium hydroxide, which enabled us to reach a final concentration of 10 mg/mL (49). Elastin was solubilized in Tris buffer 0.05 M pH 8.8 to obtain a 1 mg/mL solution. Collagen was prepared according to the procedure described by Aguilar et al. (50). Briefly, 1 mg/mL collagen stocks were prepared in 0.5M acetic acid. To generate the collagen matrix, 200 µL of stock solution was mixed with 10 µL of 10× PBS containing phenol red as a pH indicator. Using NaOH 0.5M, collagen was titrated until the red phenol turned pink. After alkaline pH, the dish containing the collagen was incubated at 37°C for 1 h to enable gelation.

#### Skin tissues

The nevus and melanoma tissues were stored in paraffin blocks. Consecutive 5 μm-thick sections were prepared for label-free analysis and hematoxylin–eosin (H&E) staining. For the label-free samples, the sections were placed on slides and heated to 60°C for 30 min in an oven to adhere to the glass. Subsequently, a drop of Canada Balsam from Biopack was applied to the sample to attach a coverslip and match the refractive index (51). For H&E staining, the sections were initially removed from the oven and cooled with xylene to remove paraffin. The samples were then returned to the oven for 30 min. Then, xylene was removed and the sections were rehydrated. Rehydration involved two dips in isopropyl alcohol, followed by two dips in 95% alcohol, and finally a dip in distilled water. Next, the sections were incubated in hematoxylin (Biopack) for 8 min, followed by a 10-minute water wash. Subsequently, the sections were incubated in yellow eosin (Biopack) for 5 min, and excess dye was washed out under running water. To complete the staining process, the sections were dehydrated with increasing concentrations of ethyl alcohol (up to 95%), followed by two dips in isopropyl alcohol. The slides were washed once with xylene and then sealed with Canada Balsam (Biopack).

#### Data acquisition

Label-free hyperspectral images from the nevus and melanoma slides were acquired using an LSM 880 laser confocal microscope (Zeiss). We used a 20× 0.5NA EC Plan-Neofluar air objective from Zeiss. Detection was performed using a Gallium-Arsenide Phosphodine (GaAsP) photomultiplier tube (PMT) set with a gain between 520 V and 550 V, collecting a spectral range from 423 nm to 723 nm in 30 steps of 10 nm each. A 405 nm laser was used for illumination, and a −405 dichroic mirror was included in the optical path to reflect the excitation light. The image size used was 1,024 × 1,024 pixels, with a pixel size of 668 nm × 668 nm, using an 8-bit dynamic range. The pixel dwell-time was 2.86 μs, and an average of two lines was used. An overlap of 5% was set for tiling to ensure data acquisition at the borders. Ten slices from patients were imaged, five intradermal nevus and five invasive melanomas. We used a 63× 1.4NA oil immersion objective EC Plan-Apochromat from Zeiss for increased resolution of HSI images of melanocytic nests. The microscope configuration (excitation wavelength, laser power, spectral range, and PMT gain) was the same as that before. The pixel size used for the ×63 objective was 132 × 132 nm, and the pixel dwell time was 0.51 μs , with four times line average. Hematoxylin–eosin images were acquired with a Motic Easyscan One commercial scanner, using a ×20 objective and 0.52 μm/pixel resolution.

#### Spectral phasor analysis

The phasor transform was used for HSI image analysis. Given an HSI stack, each pixel contains an intensity emission spectrum I(λ) as a function of wavelength (λ) (44, 52). The phasor transforms turn I(λ) into a phasor, which is a complex number that represents a sinusoidal function with a related phase and modulation. This representation can be plotted as a single point in the plane with an associated pair of Cartesian coordinates, G and S, as defined in Equations (1) and (2).


(1) $$x\;=\;G\left(\lambda \right)\;=\;\frac{\int_{\lambda min}^{\lambda max}I(\lambda )\;cos\left(\frac{2\pi n\left(\lambda -\lambda min\right)}{\lambda max\;-\;\lambda min}\right)\;d\lambda }{\int_{\lambda min}^{\lambda max}I(\lambda )\;d\lambda }$$

(2) $$y\;=\;S\left(\lambda \right)\;=\;\frac{\int_{\lambda min}^{\lambda max}I(\lambda )\;sin\left(\frac{2\pi n\left(\lambda -\lambda min\right)}{\lambda max\;-\;\lambda min}\right)\;d\lambda }{\int_{\lambda min}^{\lambda max}I(\lambda )\;d\lambda }$$

**Figure 1:**
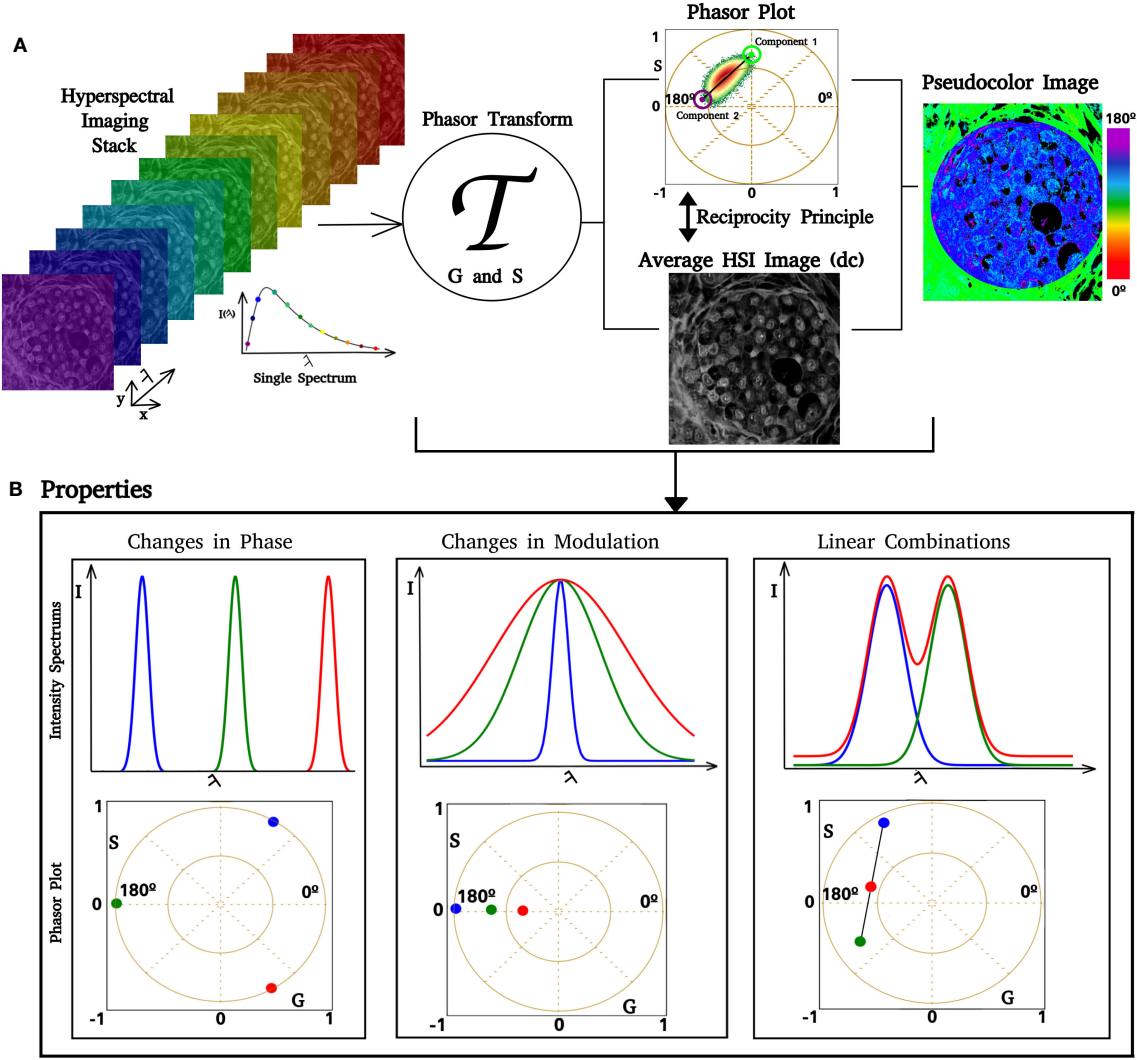
Phasor analysis pipeline. Images of increasing lambda steps from the HSI stack. If the image dimension is d=m×n , where d is a positive integer, then there are d spectra in the HSI stack. After we apply the phasor transform (represented by T  ), there are d pairs of coordinates (G, S) in the phasor plot. In **(A)**, it is possible to see that a single pixel in the lambda stack has an associated spectrum, which is transformed and represented in the phasor. The phasor plot allows us to identify how fluorescent components are distributed over the phasor space. Similar spectra have (G, S) coordinates close to each other, whereas different spectra are separated. Using the reciprocity principle, it is possible to select regions of interest (ROI) in the phasor plot, such as the blue circle, and color the related pixels of the ROI over a gray image. Selecting an ROI in the gray image also enables us to obtain phasor coordinates for this ROI. Each (G, S) pair has an associated pair of phase and modulation (ϴ, ϱ); based on that, we can create a color scale and obtain a pseudocolor image. An example is shown in the figure on the right side, where we implemented a color scale where low phases correspond to green and high phases correspond to blue. **(B)** Simulation of spectral phasor properties (changes in the spectrum center of mass, phase shift, spectrum width (modulation), and linear combination).

**Figure 2:**
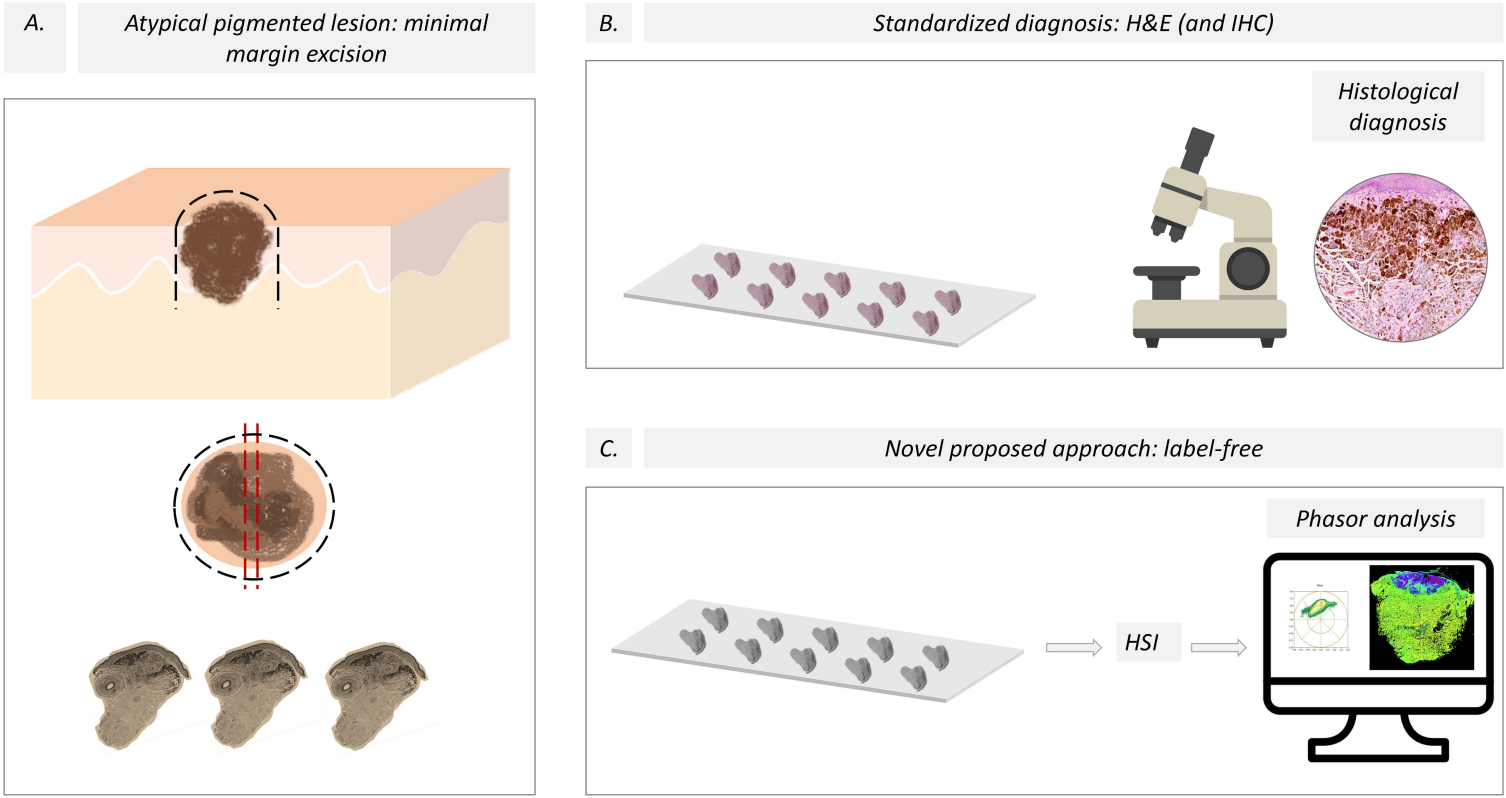
Pipeline for the evaluation of skin melanocytic lesions. **(A)** A minimal margin was used to excise the pigmented lesion, and then the sample was split using the “loaf bread” technique for the traditional and new HSI protocols. The black dashed lines represent the line of lesion excision, and the red dashed lines are the “loaf bread” cut. **(B)** The current standardized diagnosis of melanoma is based on histological studies aided by immunohistochemistry. **(C)** We propose a novel approach based on label-free slices and HSI images using spectral phasor analysis. H&E, hematoxylin and eosin; IHQ, Immunohistochemistry; HSI, Hyperspectral imaging.

Where I(λ) is the intensity at each spectrum step, λ (min) and λ (max) the beginning and the end of the spectrum, and “n” is the number of harmonic, in our case, 1. The phasor transform satisfies several properties inherited from Fourier Transform. Three are fundamental for our spectral phasor analysis: the uniqueness property, linear combination, and reciprocity principle (see **Figure 1**) (47). The uniqueness property refers to the fact that, for every spectrum, there is a unique transform and a pair of G and S in the spectral phasor plot (**Figure 1B**). This property is fundamental for two reasons: 1) it allows one to characterize and analyze every spectrum by analyzing their pair of G and S; 2) it allows one to select regions in the phasor plot according to their spectral shape because since similar spectra have their points close to each other. The linear combination property means that by having two components, any combination will fall on the line that joins the pure components (see **Figure 1B**). A second characteristic of this property enables the quantification of each component’s fraction as the distance between the position in the phasor plot from the mixture of two components and the pure components. The reciprocity principle enables the selection of regions of interest (ROI) at the spectral phasor and highlights the pixels with this selector (cursors) back to the original image (see **Figure 2A**). A pseudocolor image can be created using the phase shift of each pixel (angular shift). It is assigned a color from 0° to 180°, where 0 corresponds to red and 360 corresponds to violet. The segmentation of an ROI in the original image also provides a secondary phasor only for selected pixels. The method described above allows us to analyze, segment, and characterize regions of the sample without the need to know *a priori* the spectroscopic components. Hence, this is a label- and model-free approach.

The modulation (M) is defined as S/G and the phase (alpha) is the arctangent of (G/S). These two variables contain inherent information about the spectrum center of mass and full width at half maximum (FWHM), as shown in **Figure 1B**. The modulation retains the spectrum width and the phase retains the spectrum center of mass. The broader the spectrum, the closer to the (0,0) phasor position will appear. While the spectrum is redder, its position will appear further in the counterrevolution starting from (1,0).

All phasor transforms, denoising, tailing, and phasor-related calculations were performed using Python-based code developed by our group. The script and materials are provided in reference (53).

We used the k-means algorithm from the cluster module of the Scikit Learn Python library to obtain the cluster center of mass in the phasor plot. In our case, the sample considers the entire cluster (phasor plot) and computes the centroid, thus minimizing the inertia of the distribution (54). The implementation of these methods is presented in (53).

### Statistical analysis

The nevus and melanoma slices were compared by measuring the center of mass for the modulation and phase histograms. A dermatopathologist defined the region of interest for quantitative comparison. The center of mass of the phase and modulation histograms were calculated using Equation (3), where f(x) corresponds to the data distribution.


(3) $$CM=\frac{{\;}^{\sum }\;f\left(x\right)\;x}{{\;}^{\sum }\;f\left(x\right)}\;$$

A T-test was performed to evaluate the relationship between the modulation and phase groups for nevus and melanomas. A p-value of 0.01 was considered the threshold to refuse or not the null hypothesis. It was calculated in Python 3.10, using the ttest_ind function of the scipy.stats module. A graph of modulation vs. phase was constructed to cluster the data. A confidence ellipse was then obtained for the distribution of melanomas and nevi. Three confidence ellipses were built considering a standard deviation of *σ* = 68.5%, 2*σ* = 95.5%, and 3*σ* = 99.7%, corresponding to the standard deviation used to build the ellipses’ axis.

## Results

**Figure 3:**
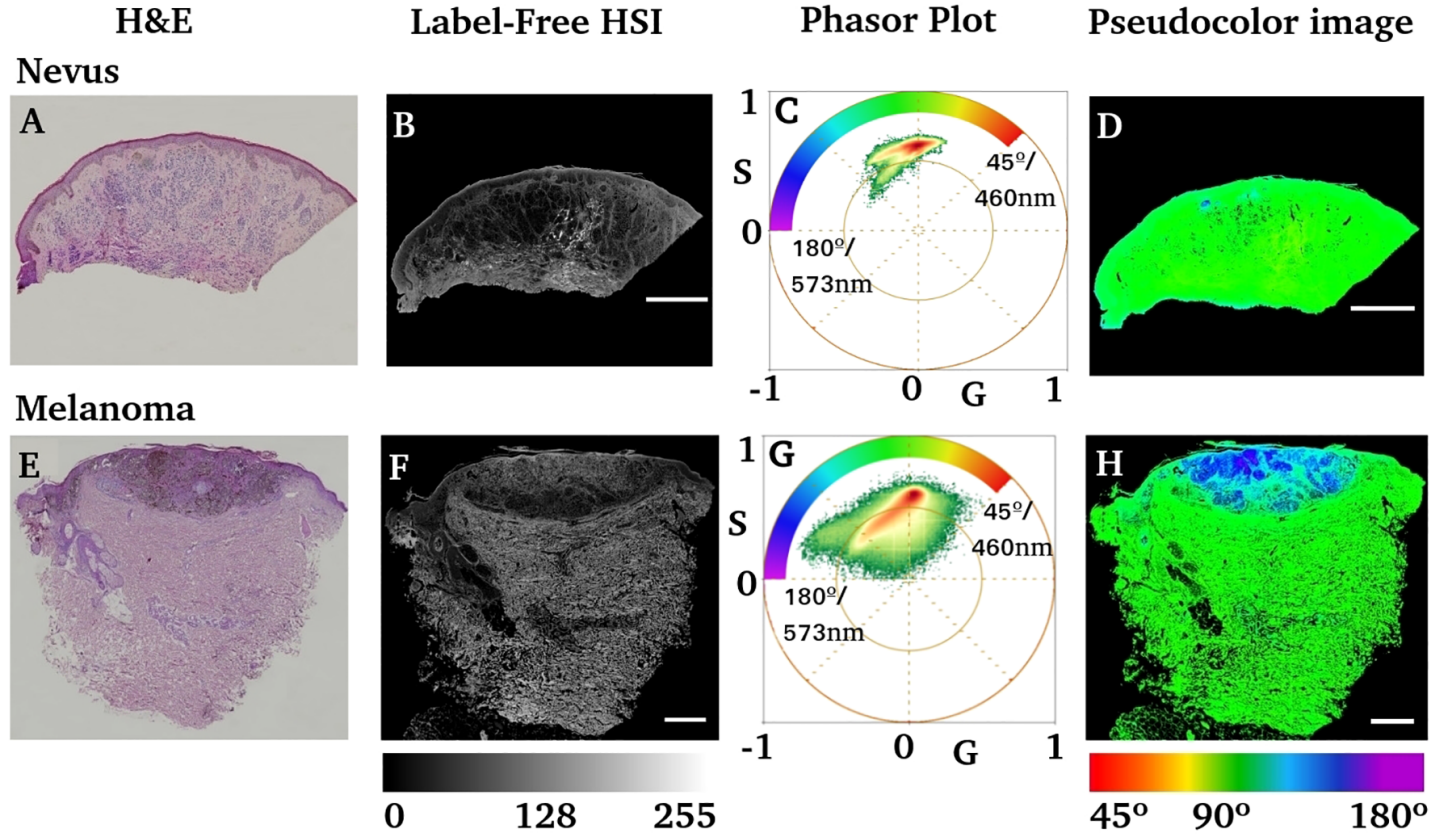
Comparison of nevus and melanoma anatomo-pathology using H&E and label-free HIS-phasor plots. **(A, E)** Hematoxylin–eosin images of the nevus and invasive melanoma samples. **(B, F)** Label-free average intensity of HSI obtained from the previous two tissue blocks (A, E, respectively). **(C, G)** Phasor plots obtained from the HSI images in **(B, F)** respectively. The inserted color scale represents the color scheme used to generate a pseudocolor image based on the phase of each pixel. **(D, H)** Pseudocolor images obtained by using the color scale shown in **(C, G)**. Scale bars represent 500 μm .

**Figure 3A** shows the hematoxylin–eosin images of an intradermal nevus and an invasive melanoma (**Figure 3E**), which contain the injured regions formed by melanocytes. The label-free HSI images in **Figures 3B, F** show the entire sample; the scale bars represent 500 μm . The tiles of the lambda stacks form these images. **Figures 3C, G** show the phasor plots obtained for both samples. A color scale was created to illustrate the phase changes in the phasor distribution, ranging from 45° to 180°, corresponding to 460 nm to 573 nm, related to the spectral center of mass. The spectral phasor showed differential spectral components compared to the nevus and melanoma. Note that the phasor-plot distribution from the nevus presented two main trajectories, whose components ranged from 75° to 135°. The calibration od the wavelength to the phase is provided in **Figure S1**.

However, the phasor distribution obtained for melanoma (**Figure 3G**) is different from that of the nevus. The phase range starts from 45° to 180°, indicating a redder component on its phasor, corresponding to a fluorescent emitter with a wavelength beyond 573 nm. The pseudocolor image obtained by the phase (**Figures 3D, H**) shows a bluish region of interest in melanoma slides that coincides with the H&E purple region identified as the lesion by an expert dermatologist.

**Figure 4:**
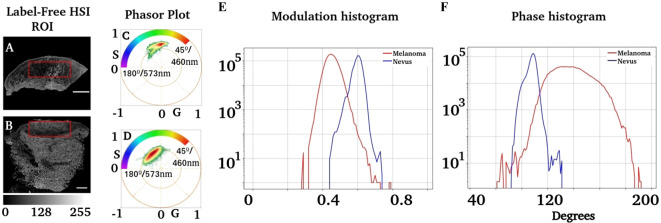
Analysis of phasor distribution in the region of interest associated with injuries. **(A, B)** Regions segmented where melanocytic injuries were present. An expert dermatologist identified the regions of interest. **(C, D)** Phasor plots corresponding to these regions. **(E)** shows the modulation distribution for the nevus and melanoma regions, whereas **(F)** represents the distribution of the phase.

**Figure 5:**
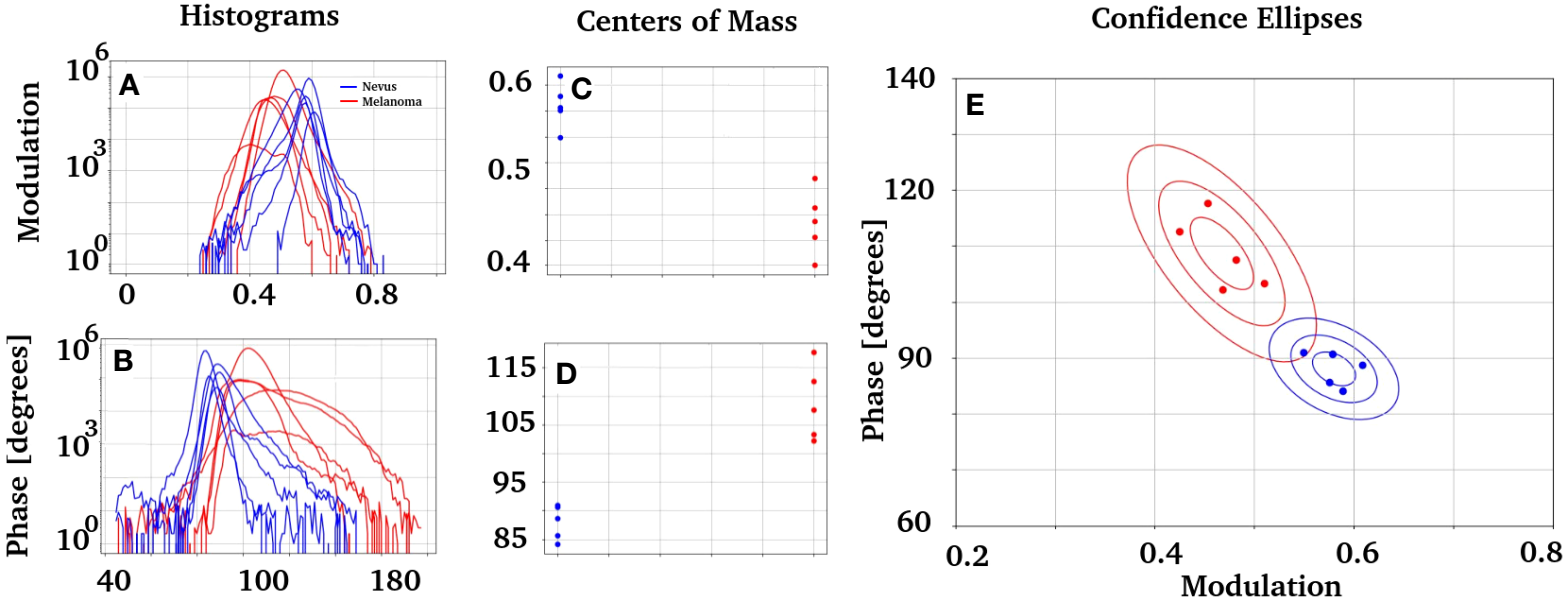
Study of the modulation and phase distribution obtained from the patients in **Table 1** with intradermal nevi or invasive melanomas. **(A, B)** Histograms of modulation and phase, respectively, for nevus and melanomas. **(C, D)** present the center of mass of each distribution. Those groups are statistically described with the average and standard deviation as follows: the nevi group has x = 0.47 ± 0.03 for the modulation and x = 108 ± 6 for the phase. In comparison, the melanoma group has x = 0.58 ± 0.02 for the modulation and x = 88 ± 3 for the phase. A p = 0.0003 was obtained for the modulation group, while p = 0.0008 was obtained for the phase group. **(E)** presents two groups of data obtained when plotting the modulation vs. phase. It also has the confidence ellipses obtained for each group, built with a standard deviation of *σ* = 68.5%, 2*σ* = 95.5%, and 3*σ* = 99.7% each one.

An analysis of the phase and modulation variables from the region of injury for both cases is shown in **Figure 4**. The phasor plot contains only the spectral components for the region of interest identified in **Figures 4A, B**. Notice that both phasor distributions are significantly different, while **Figure 4C** presents the main component at 90° (498 nm), and the melanoma distribution was widely distributed, ranging from 90° to almost 180° (498 nm to 573 nm). The modulation and phase histograms for each distribution are plotted separately (**Figures 4E, F**). The phase and modulation quantification for the ROI showed that the melanoma presents a higher phase component that does not exist in the nevus. **Figure 5** was constructed using the samples listed in **Table 1**. Note that the images for all sample studies can be found in **Figures S2–S11** in the upplementary material. Half of the patients were diagnosed with intradermal nevus, and the other half with invasive melanoma. **Figures 5A, B** showed more heterogeneous results than those described previously. To quantify the many samples, we calculated the center of mass for both magnitudes (modulation and phase histograms) (**Figures 5C, D**). The nevi group had an average and standard deviation of 0.47 ± 0.03 for the modulation and 108 ± 6 for the phase. In contrast, the melanoma group had a modulation of 0.58 ± 0.02 and 88 ± 3 for the phase. The p-value for the modulation group was 0.0003, whereas that for the phase group was 0.0008. Finally, we generated a scatter plot combining both magnitudes to generate a histogram comparison of these numbers (**Figure 5E**).

The confidence ellipses in E have a standard deviation of *σ* = 68.5%, 2*σ* = 95.5% and 3*σ* = 99.7%. There is a solid difference between the nevus and melanoma phasor components. It would be interesting to compare the phasor fingerprints of nevus and melanoma with those of the normal skin. However, using normal skin for this purpose is ethically unjustifiable. We analyzed “healthy” ROIs at the nevus slices to characterize their autofluorescence fingerprints to overcome this limitation. We analyzed ROIs from different nevi and calculated each sample’s modulation and phase histograms (**Figures S12–S15**). The results indficated a similar phase and modulation distribution in the nevus tissue.

**Figure 6:**
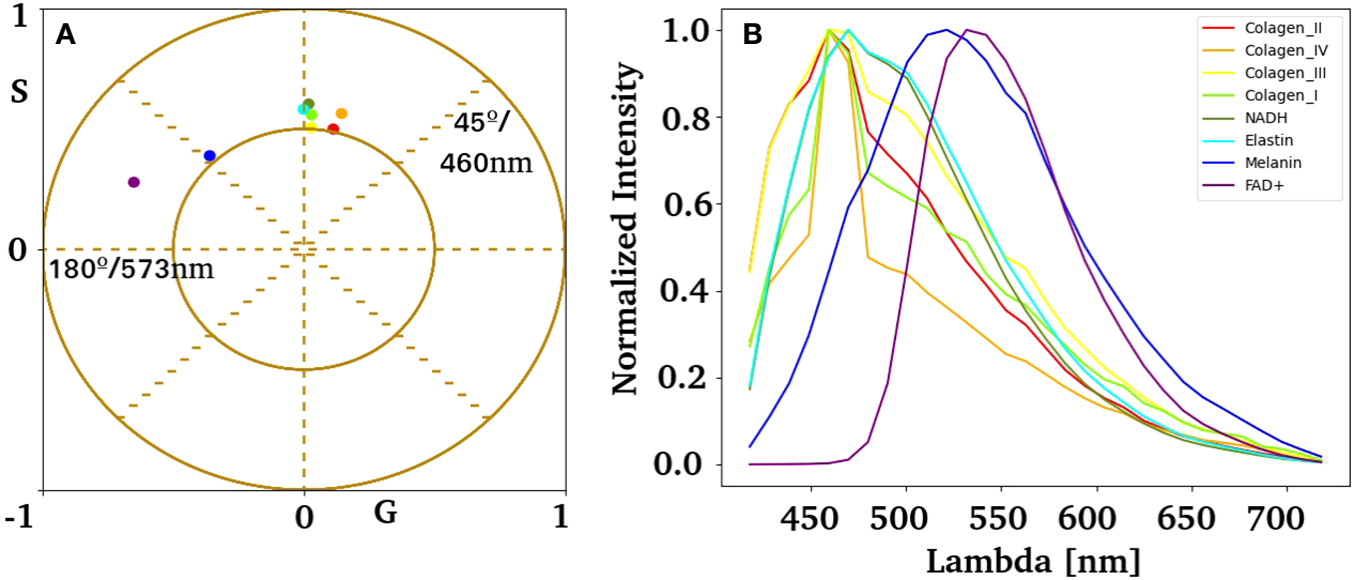
Spectral phasor fingerprints for autofluorescent biomolecules in the skin. **(A)** Pure component centroid obtained with the k-means algorithm using HSI data acquired experimentally from independent samples listed in **(B)**. **(B)** Average emission spectrum for collagen I, II, III, IV, elastin, NADH, FAD, and melanin.

We obtained the HSI of the autofluorescent molecules described in the skin to identify the autofluorescent component responsible for the nevus and melanoma phasor plot clusters. **Figure 6** shows the phasor position for the most abundant fluorophores in the skin, namely collagen, elastin, NADH, FAD, and melanin. Note that collagens have similar spectral fingerprints (and are bluer fluorescence), and NADH and elastin are greener. The two autofluorescent molecules responsible for the redder fingerprint are FAD+ and melanin. Interestingly, all autofluorescent biomolecules studied showed unique fingerprints that could be identified in the nevus and melanoma samples.

**Figure 7:**
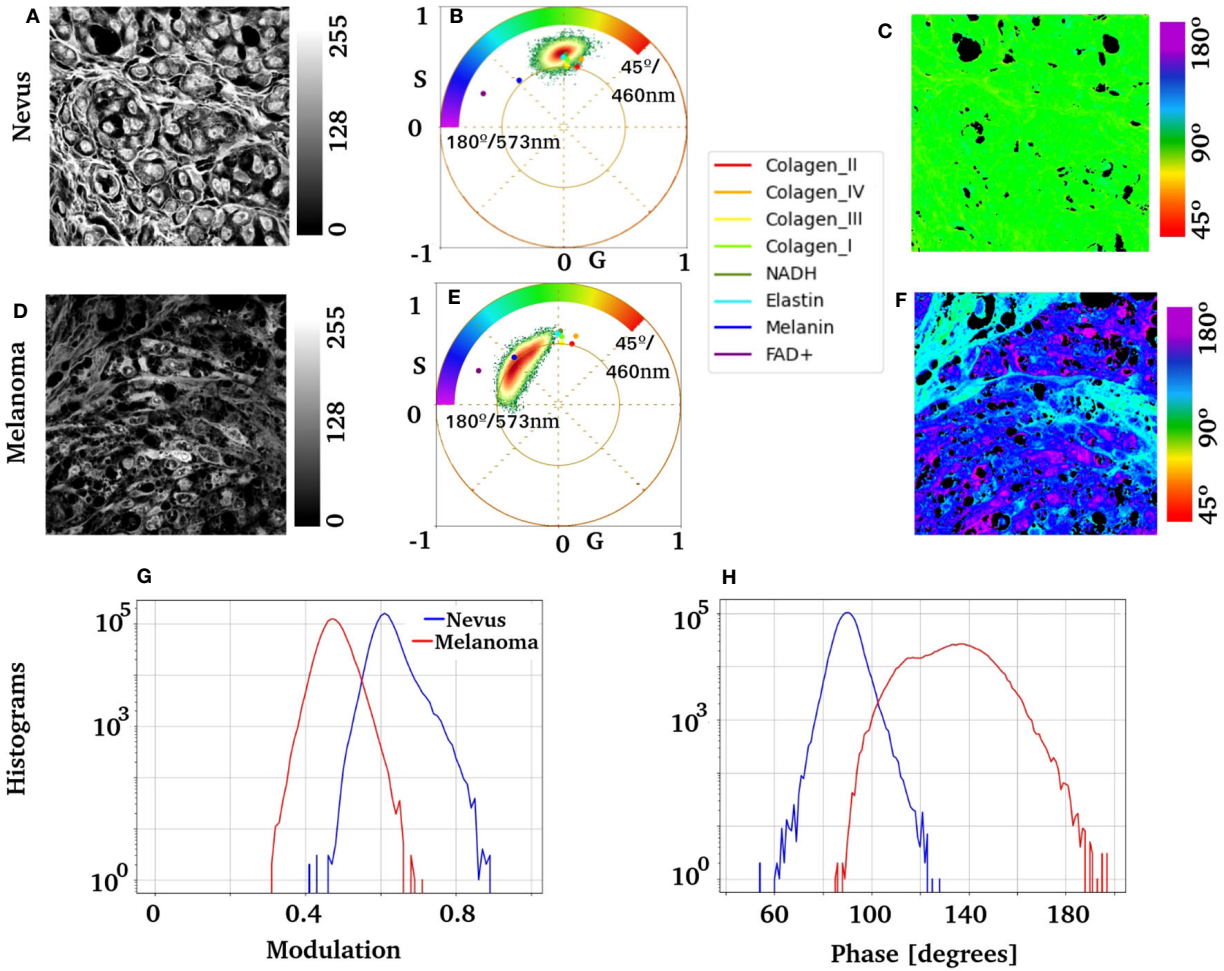
Nests of melanocytes were imaged at ×63 from a nevus and an invasive melanoma. It shows the phasor analysis for these regions and the distribution of the previously obtained pure components. **(A, D)** Images show the label-free average intensity image of the HSI stack; it can appreciate nests of benign and atypical melanocytes, respectively. Images **(B, E)** show the corresponding phasor plot and the centroid of each molecular fingerprint overlaid. Scale bars are 50µm . **(C, F)** present pseudocolor images of the nevus and melanoma, respectively. **(G)** shows the modulation histogram, and **(H)** shows the phase histogram for both samples.

**Figure 7** shows high-resolution images of the nevus and melanoma nests. To identify autofluorescence in nevus and melanoma nests, the centroid of the pure components obtained in **Figure 6** was overlaid with the phasor distribution from the tissue. Note that both clusters overlap with the species studies. NADH, collagen, and elastin dominate the nevus fluorescence fingerprints. In contrast, melanoma showed strong linear combinations of FAD and melanin. However, the redder component in melanoma remains elusive to the molecules studied here. The histograms for phase and modulation of the nests showed substantial differences between the nevus and melanoma (**Figures 7G, H**).

## Discussion

Melanoma is a malignant neoplasm that originates from melanocytes (15). If an early diagnosis is missed, its aggressiveness and metastatic potential result in a high mortality rate (37). Diagnosis relies on conventional methods, primarily histopathology, based on architectural and cytological criteria (2). Recent studies have revealed an important issue in melanoma diagnosis owing to discord among experts, with up to a 25% difference in opinion when distinguishing between melanoma and nevi through histological examination (55). In suspicious cases, additional immunohistochemical stains are typically requested; nonetheless, no single method currently can confirm or rule out melanoma. Therefore, there is a particular interest among researchers in developing new diagnostic tools to assess this diagnostic problem and define the boundaries between melanomas and nevi (16, 56).

The use of novel techniques, such as multiphoton and FLIM, demonstrates enormous potential for the quantitative assessment of skin lesions (31). However, these technologies are expensive and require expertise to realize their full potential. This study aims to offer a new toolkit that relies on commercial microscopes using single-photon excitation and a method for analysis that does not require an *a priori* assumption as to which endogenous fluorescent molecules are present in the tissue. Hyperspectral imaging using confocal microscopes has been widely implemented in many commercial brands. Therefore, it is a great tool for approaching autofluorescence from unlabeled skin slides.

Spectral phasor analysis of HSI data from melanoma regions demonstrated the occurrence of a molecular fingerprint due to the specific biochemical composition of degenerated melanocytes (**Figures 3**, **4**). The use of autofluorescence fingerprints has limited relations with the metabolic states of fresh tissue, and different fixation procedures can modify the outcome obtained by the phasor plot (32, 33). However, there was value in the remaining tissue autofluorescence to discriminate between nevus and melanoma (**Figure 5E**). The evaluation of melanoma versus nevus phasor modulation and phase center of mass showed a 95.5% confidence interval when these two groups were compared. The molecular fingerprint identified in each group showed robust characteristics, such as increasing phase (longer wavelength fluorescence molecules) and smaller modulation, indicating a strong influence of redder components partially identified as FAD and Melanin (**Figures 4**, **7**). However, melanoma tissue has a strong shift to longer wavelengths that move the phasor trajectory further than melanin used as a standard (**Figure 7E**). One possible explanation is the existence of different amounts of eumelanin and pheomelanin, which have different fluorescence lifetimes and spectral emissions (57–59). These considerations point to eumelanin as the longer-wavelength component with an approximately broad maximum at 640 to 680 nm, and pheomelanin emission peaks around 615 nm to 625 nm (57). A previous study by Fereidouni et al. described the value of spectral information to identify key molecular fingerprints within the skin (44). However, multiphoton excitation was used in this case, which, while more appropriate for broad excitation of autofluorescence, limits the adoption of this technology in clinical environments. UV excitation is limited by its penetration and capability to excite a wide range of autofluorescent molecules (60). Our rationale for the use of 405 nm excitation was to answer a simple question. Was there any value in the autofluorescence fingerprints from tissue at this excitation to develop a method that can be adopted by any user with access to a regular confocal microscope with standard laser lines? The 405 nm excitation is a valuable wavelength for the excitation of most endogenous autofluorescent molecules and does not require expensive lasers such as multiphoton systems. The broad phasor cluster obtained in our melanocytic samples supports the value of 405 nm excitation, showing its capability to separate melanoma and nevus (**Figure 3**). Other single-photon excitation lines, such as 440 or 488, can be added to provide novel phasor fingerprints with longer acquisition times.

To compare fingerprints obtained from nevus and melanoma with normal skin, phasor plots from “healthy ROIs” in the nevus slices were analyzed (see **Figures S12–S15**). ROI selection was performed by our anatomopathology experts. Interestingly, the fingerprints are similar to those found in the nevus, confirming the distinctive fingerprint of malignant melanocytes in melanoma versus benign nevus. The same experiment cannot be extrapolated to melanoma lesions because of dermal modification caused by the tissue during melanoma progression.

Phasor differences between melanoma and nevus also showed a substantial contribution from NADH and FAD fluorescence. The FAD/NADH ratio has been used as a metabolic index in *in vivo* experiments (61). In our fixed tissue, the metabolic significance of this ratio was poor; nonetheless, this fingerprint can reflect a picture of the pre-fixation melanoma metabolic cellular state. Interestingly, malignant and dysplastic lesions display differences in histological appearance and biological characteristics (62). Carcinogenesis involves a dedifferentiation and transformation process that enables the proliferation of genetically unstable cells. Even primary melanomas exhibit extreme heterogeneity in cell subsets and mutational profiles (63, 64). Melanoma metabolic rewiring and phenotype-switching are interconnected events that dictate the tumor microenvironment distribution of endogenous molecules, such as fluorophores (64). This literature supports the idea of a melanoma autofluorescence fingerprint judged by its spectral phasor plot signature, which can relate to the metabolic shift in the lesion.

Hyperspectral imaging and phasor analysis of the tissues examined (10 melanocytic lesions, five of which were benign and five were malignant) provided positive values for quantitative assessment of the correlative evaluation of the phase and modulation center of mass (**Figure 5E**). Despite the relatively small sample size, this study identified a robust statistically significant difference in modulation and phase between both groups, indicating that this analysis is a potentially reliable indicator for differentiating between malignant and benign melanocytic proliferation. The most remarkable feature of this approach is the opportunity to support dermatopathologists with numbers based on molecular fingerprints, which does not require individual training, such as in H&E histopathological analysis.

Introducing the HSI-phasor-based melanoma diagnosis (**Figure 2C**) into the dermatologist workflow (**Figure 2B**) should be feasible because of the low complexity of the instrumentation and analysis required. In addition, preparing label-free paired slides for regular H&E staining will not affect the standard dermatology procedures but will add a new quantitative layer to the anatomopathology to support their sample report. The lack of laser scanning microscopy in hospitals or dermatology clinics could be a limitation. However, this issue can be overcome by preparing samples to be sent to a confocal microscope center where HSI can be performed. Phasor plot analysis may be an obstacle to adopting our approach in the dermatology department.

Nevertheless, we believe that the experience gained from remote digital pathology diagnosis (65) can be applied to this approach. Our straightforward analysis can be performed remotely, providing results using simple and quantitative numbers. This approach avoids the need for complex interpretations that require in-house expertise in spectroscopy and phasor analyses.

Our method has some technical limitations that need to be addressed. Owing to the large average size of skin lesions, using a laser scanning microscope requires tiling to cover the whole slice. Spectral collection can extend the imaging time by the number of spectral steps defined if the spectral configuration of the microscope is used. As in our case, the spectrum was collected in 30 steps, increasing the time needed for the complete x/y/lambda acquisition by 30 times. However, spectral detectors with an array or spectral camera can speed up acquisition time. To reduce the acquisition time, we rely on averaging twice the spectrum in each pixel and using the median filter at the phasor to decrease phasor noise and improve phasor cluster distribution. The average time for HSI in our samples was around 2 h–3 h; however, considering that there is no urgency for melanoma anatomo-pathology diagnosis, as there is in carcinoma assessment during Mohs surgery, this approach matches the average time for H&E staining. In contrast, the HSI acquisition and phasor analysis are fully automated and can be run in parallel with H&E preparation and observation.

## Conclusion

Our results demonstrate the value of HSI in combination with spectral phasor analysis for label-free identification and quantification of melanomas. This technology can support dermatologists in skin diagnosis, in addition to the traditional H&E quantitative information from molecular autofluorescent fingerprints in melanocytes from melanoma. Further research is needed to understand their value in the stratification of different melanoma and nevi lesions.

## Data availability statement

The raw data supporting the conclusions of this article will be made available by the authors, without undue reservation.

## Ethics statement

The studies involving humans were approved by Prof. Dr. Raúl Ruggia, Coordinator of the Research Ethical Committee at the Hospital de Clinicas, Universidad de la Republica, Uruguay. The studies were conducted in accordance with the local legislation and institutional requirements. The participants provided their written informed consent to participate in this study.

## Author contributions

BS: Formal Analysis, Investigation, Methodology, Resources, Software, Visualization, Writing – original draft. SM: Formal Analysis, Investigation, Methodology, Resources, Writing – original draft. AG: Methodology, Supervision, Writing – review & editing. FL: Formal Analysis, Software, Writing – review & editing. JM: Conceptualization, Methodology, Resources, Writing – review & editing. LM: Conceptualization, Data curation, Funding acquisition, Methodology, Project administration, Supervision, Writing – review & editing.
