# Supplementary material for: Quantitative melanoma diagnosis using spectral phasor analysis of hyperspectral imaging from label-free slices
Source: Front Oncol. 2023 Nov 17;13:1296826. doi: 10.3389/fonc.2023.1296826 (PMC10756080; doi:10.3389/fonc.2023.1296826)
Supplement: Supplementary file 1 [file DataSheet_1.docx]

## **Supplementary Material**

## **Quantitative melanoma diagnosis using Spectral Phasor Analysis of Hyperspectral Imaging from label-free slices**

**Data and simulations:**


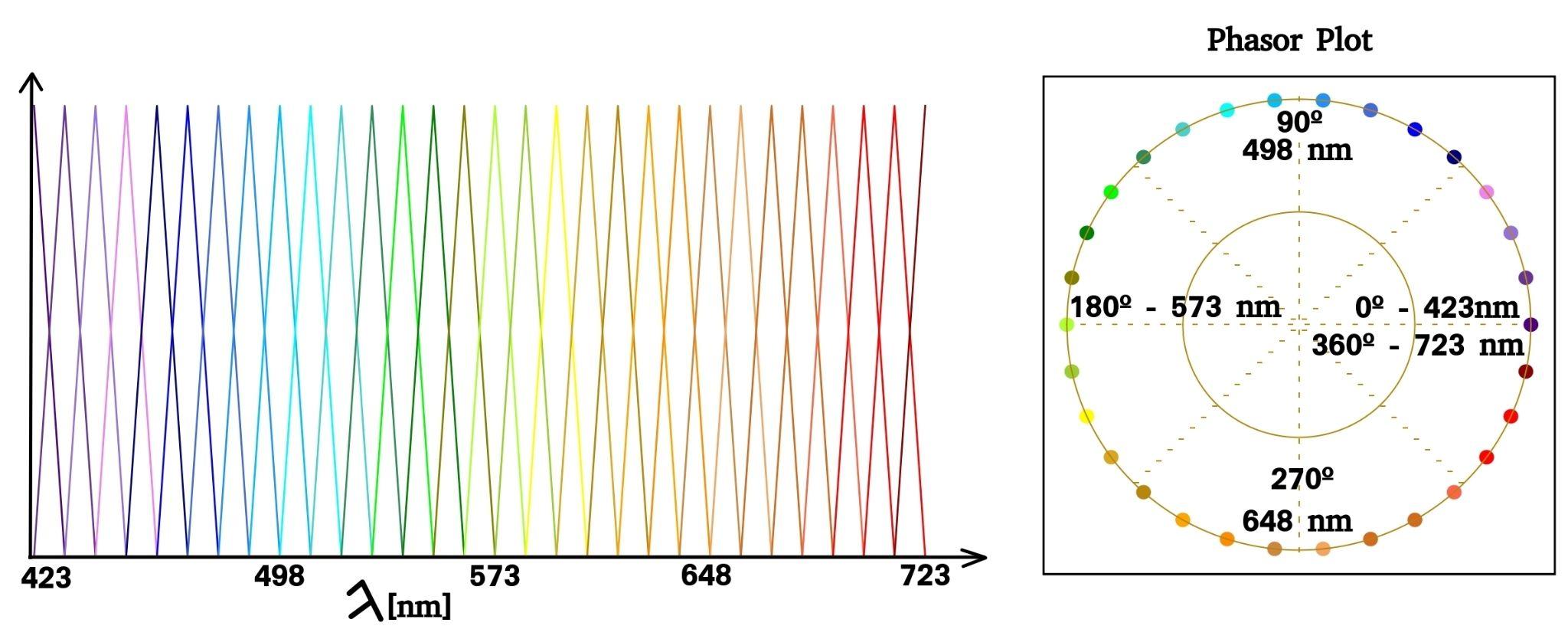


**Figure S1:** Spectra simulation to calibrate the wavelength in correlation with the phase range along the phasor plot. Thirty sharp spectra were simulated with the same FWHM but shifted maximum across the entire spectral range in our HSI configuration (423-723 nm). Notice that the position at the phasor plot does not reflect the spectrum maxima but rather the spectrum center of mass as defined in the G and S calculation.

## **Intensity fluorescence images, phasor plot information, and pseudocolor images obtained from each lesion studied.**


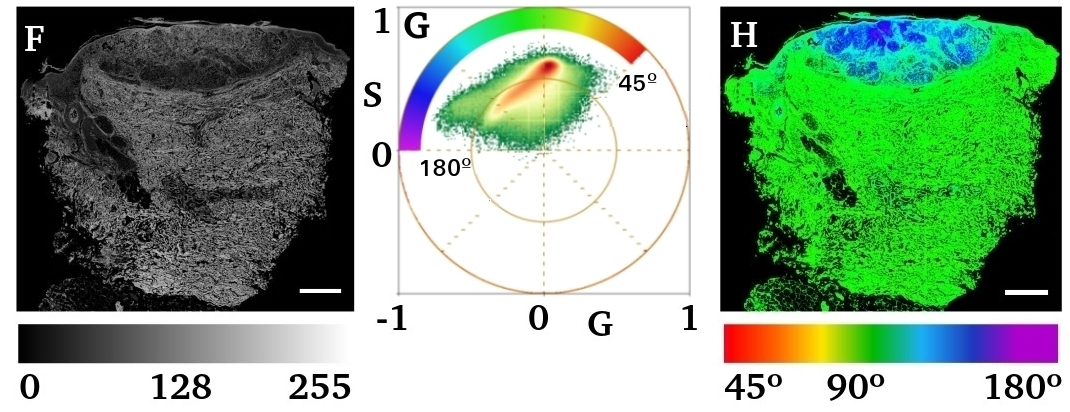


**Figure S2:** Invasive Melanoma Breslow 0.9 mm (in table IM1).


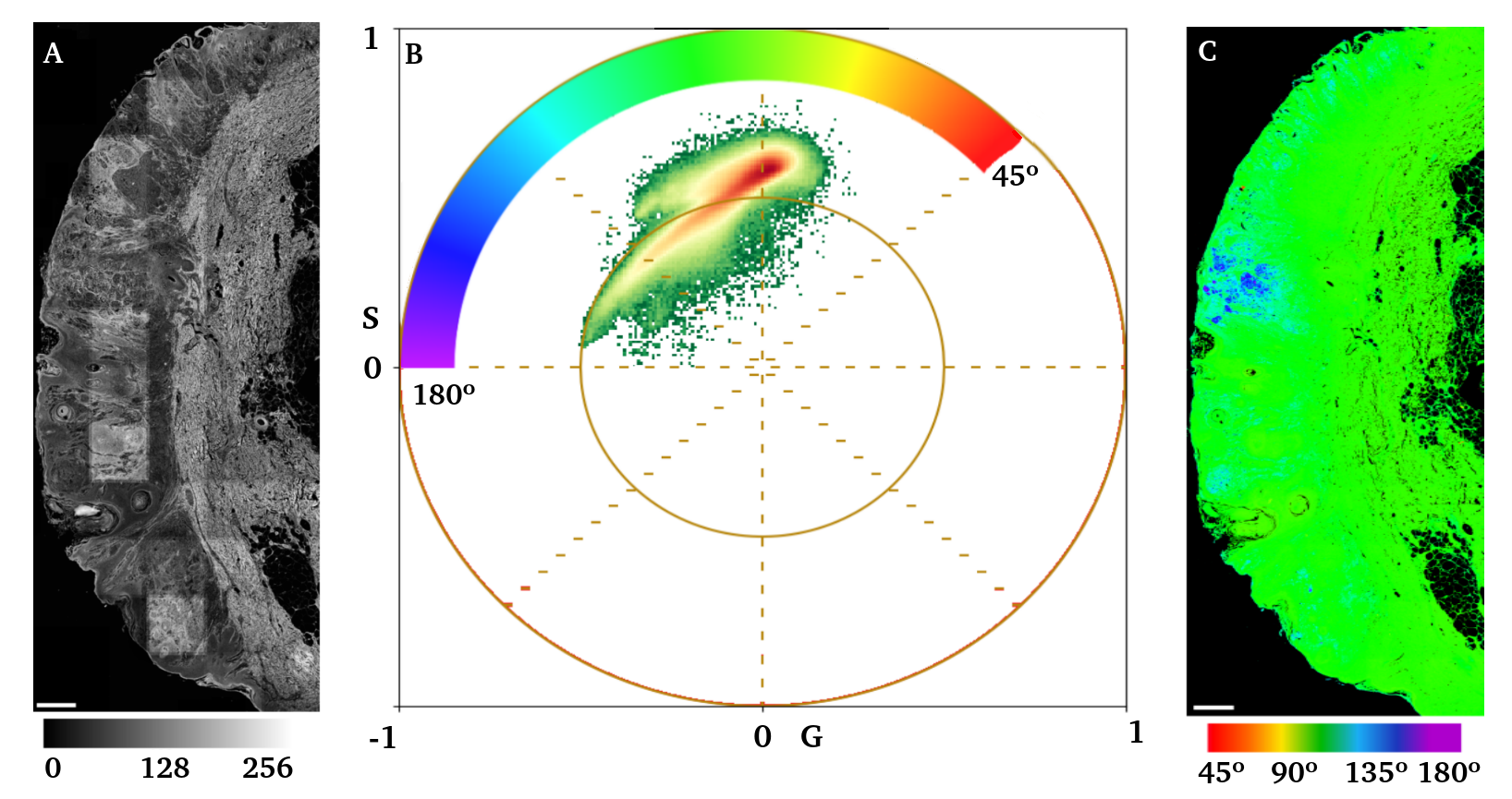


**Figure S3:** Invasive Melanoma Breslow 1.4 mm (in table IM2).


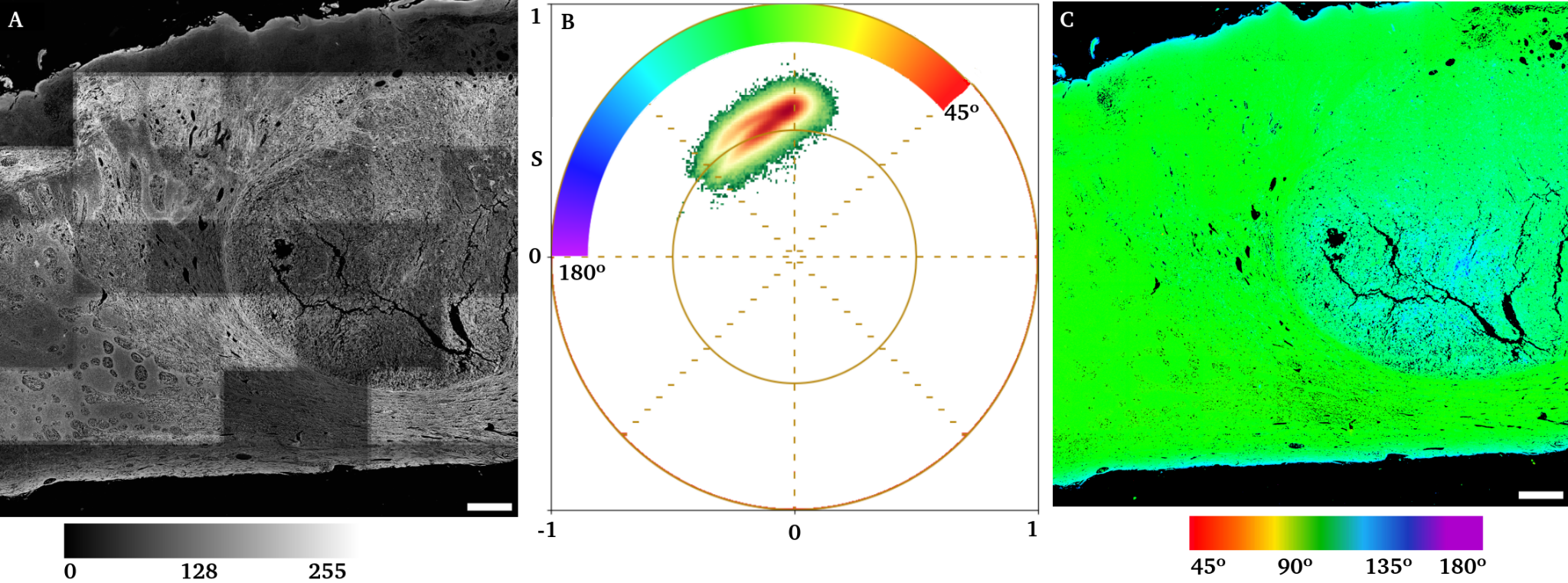


**Figure S4:** Invasive Melanoma Breslow 6 mm (in table IM3).


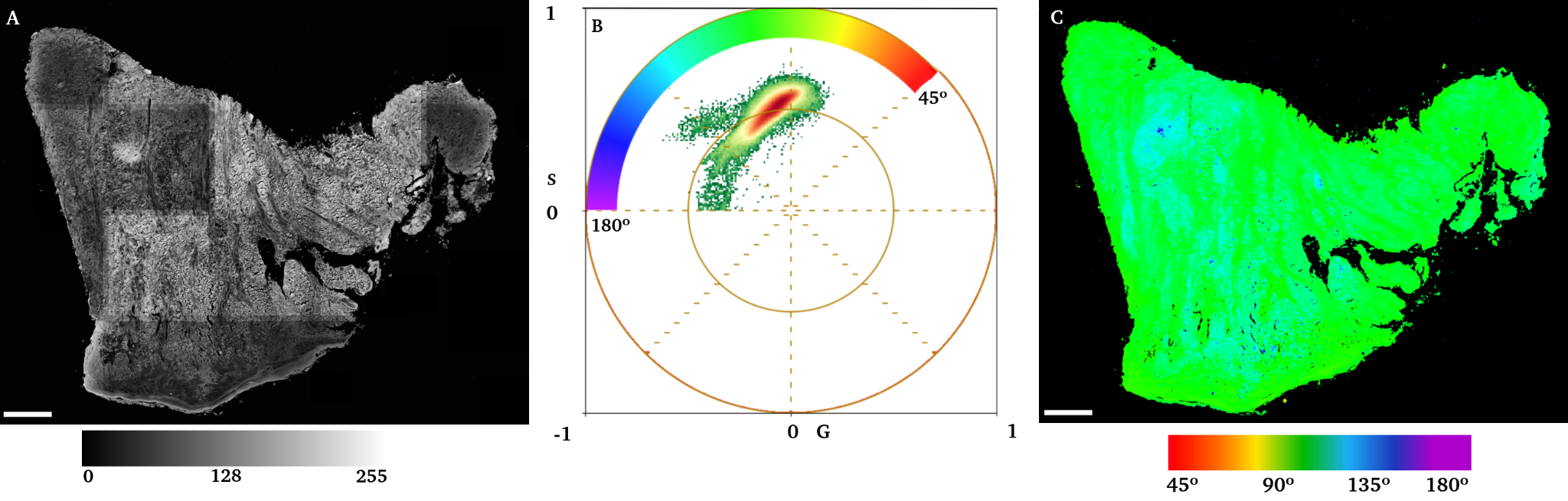


**Figure S5:** Invasive Melanoma Breslow 2.5 mm (in table IM4).


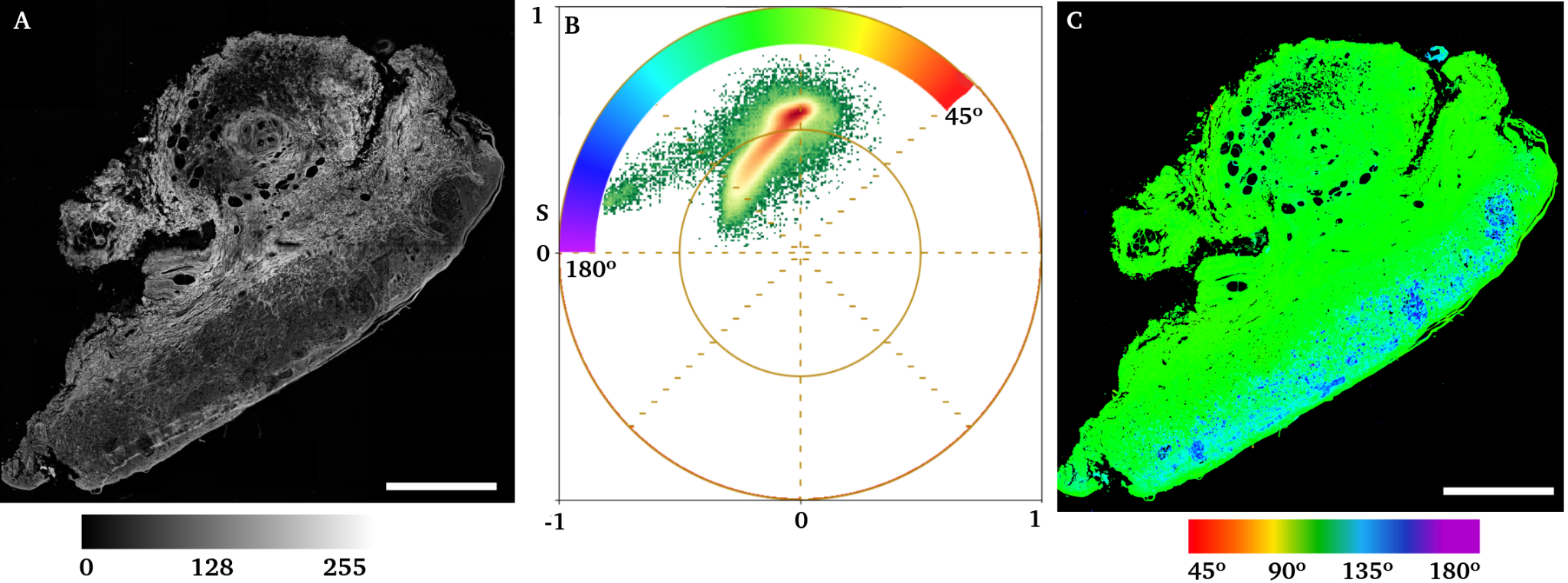


**Figure S6:** Invasive Melanoma Breslow 0.5 mm (in table IM5).


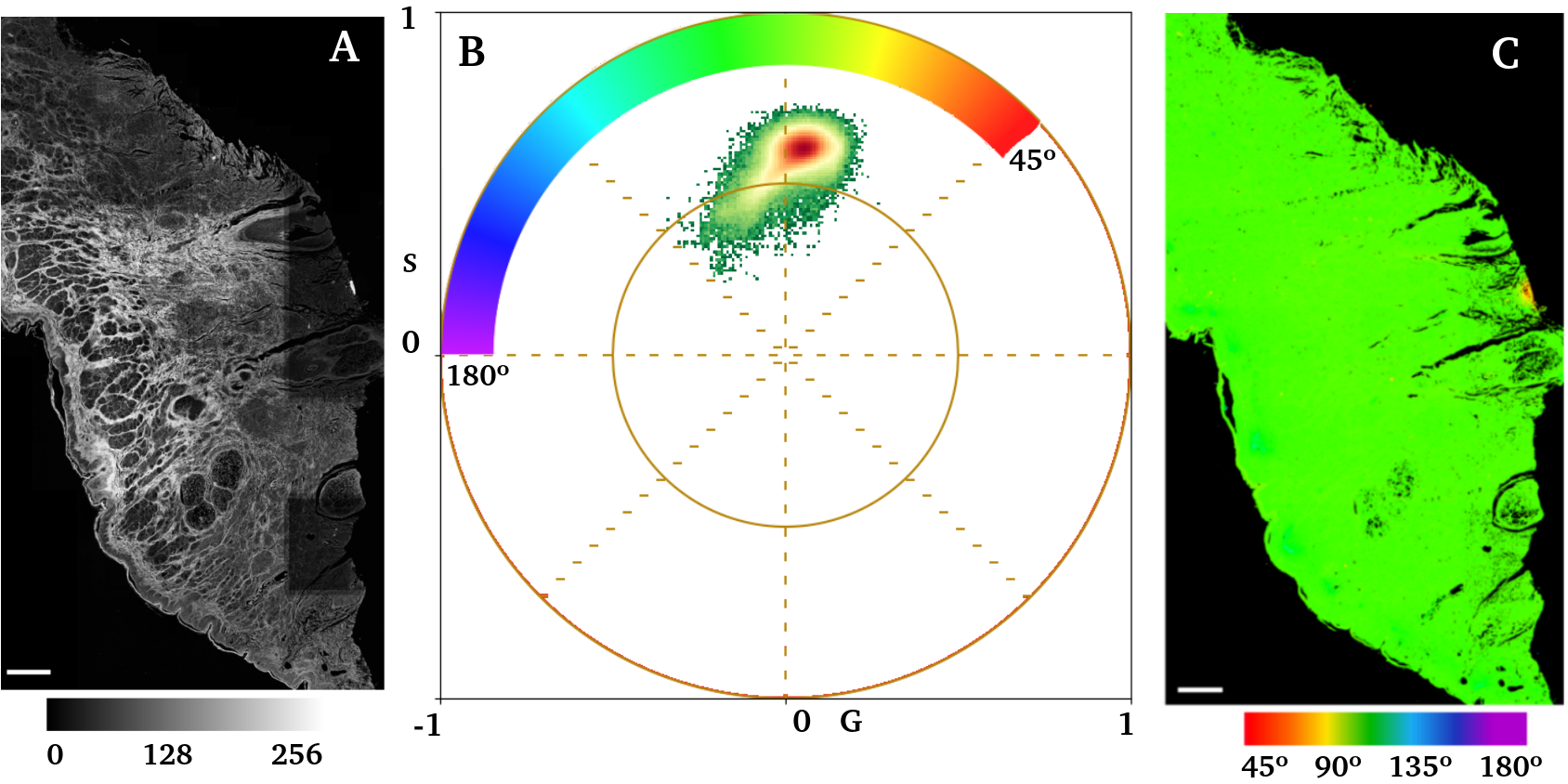


**Figure S7:** Intradermal nevus IN1.


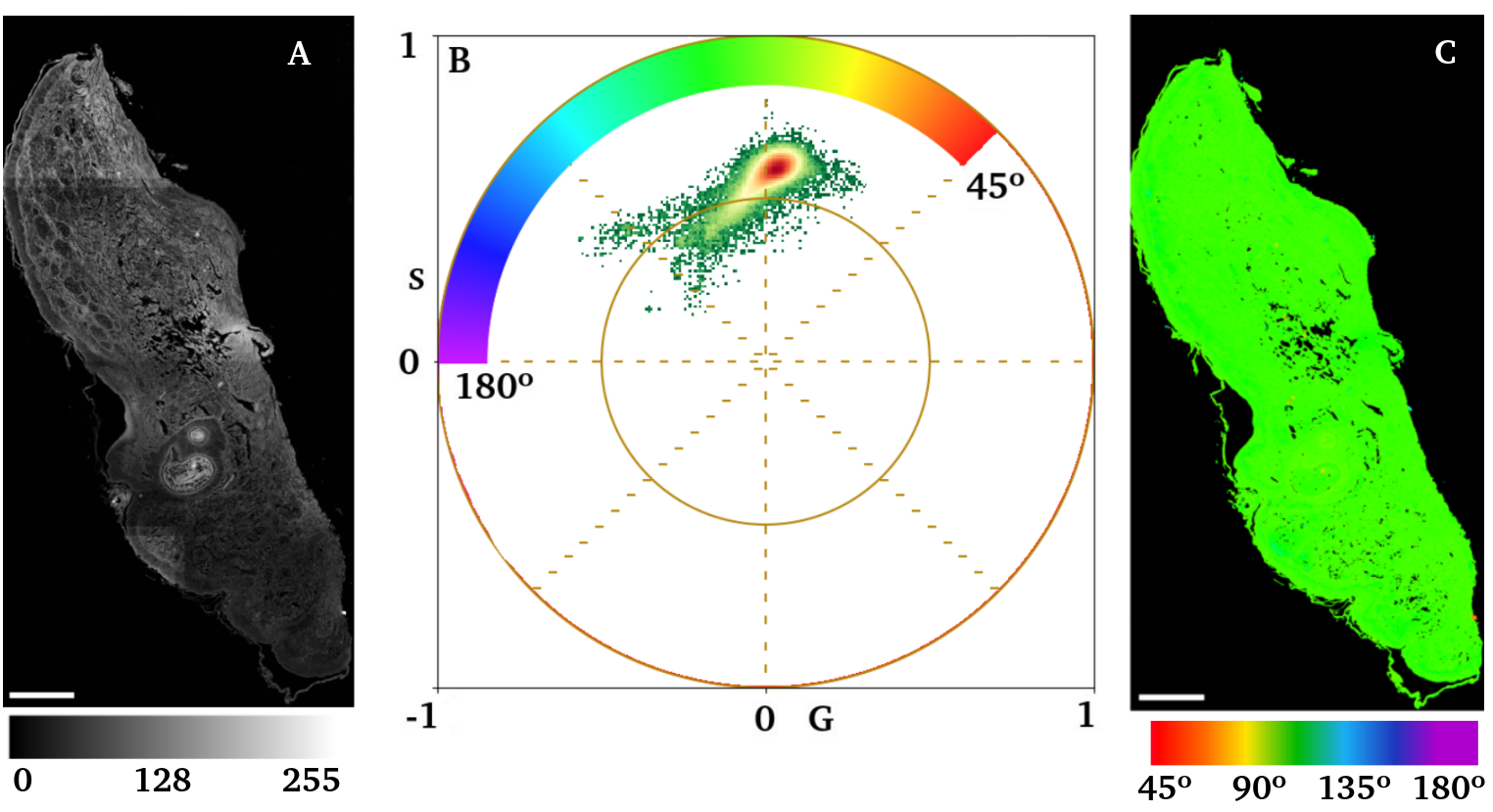


**Figure S8:** Intradermal nevus IN2.


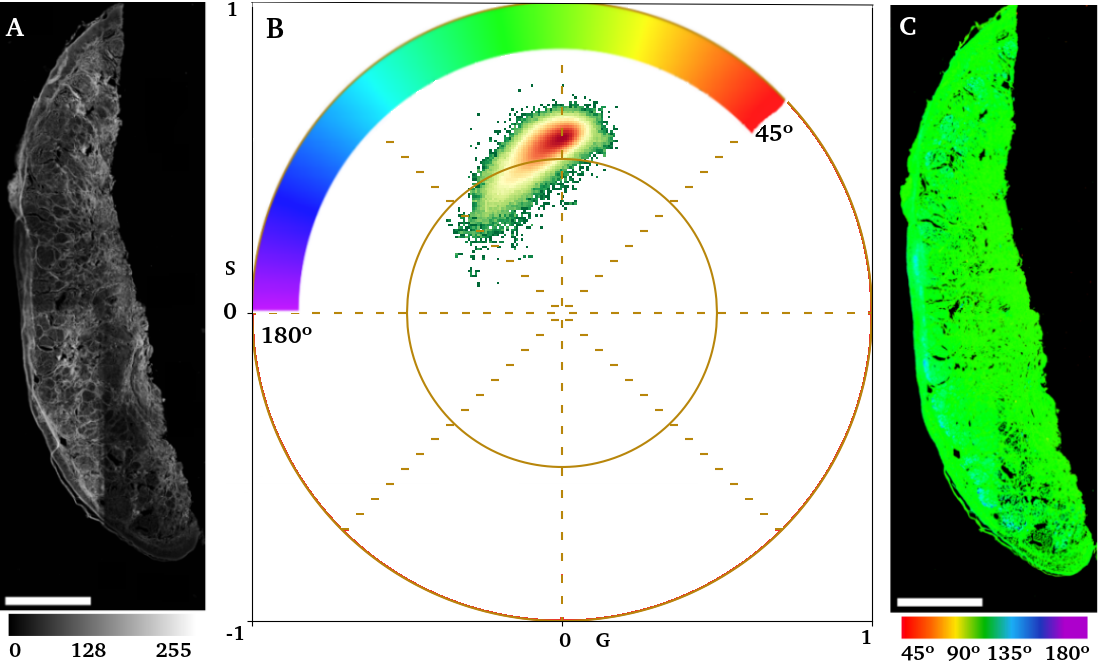


**Figure S9:** Intradermal nevus IN3.


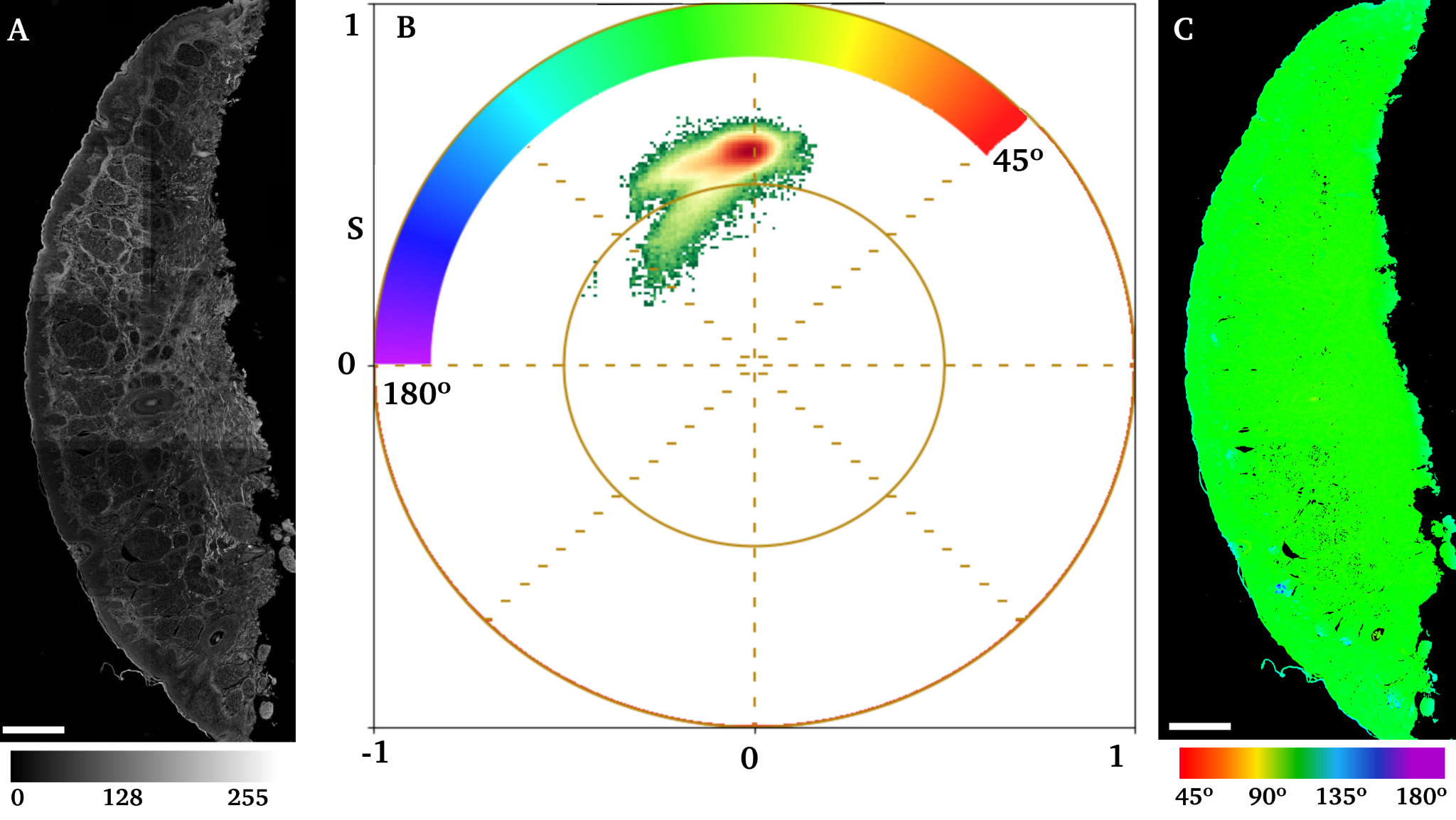


**Figure S10:** Intradermal nevus IN4.


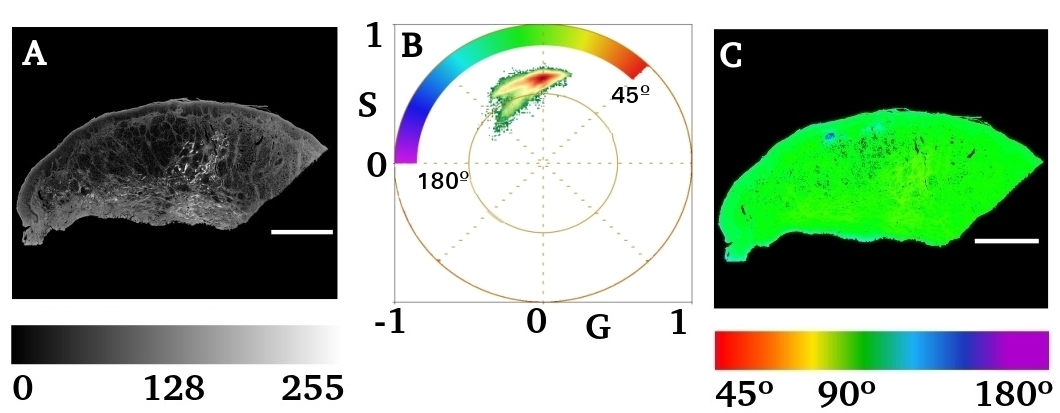
**Figure S11:** Intradermal nevus IN5.


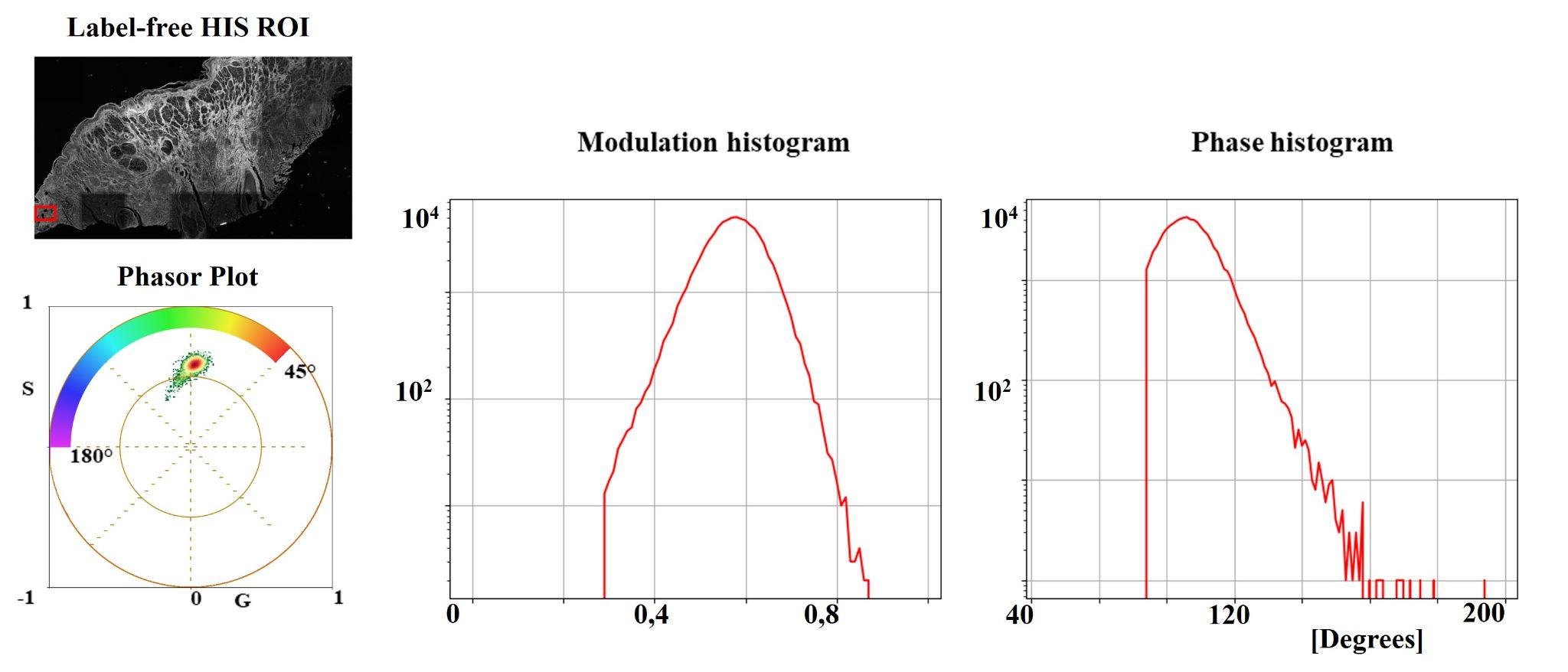


**Figure S12: Analysis of Phase and Modulation profile for ROI in “normal” tissue outside the lesion.** ROI for IN1 with non-melanocytic lesion, its associated phasor plot, and its modulation and phase histograms.

##
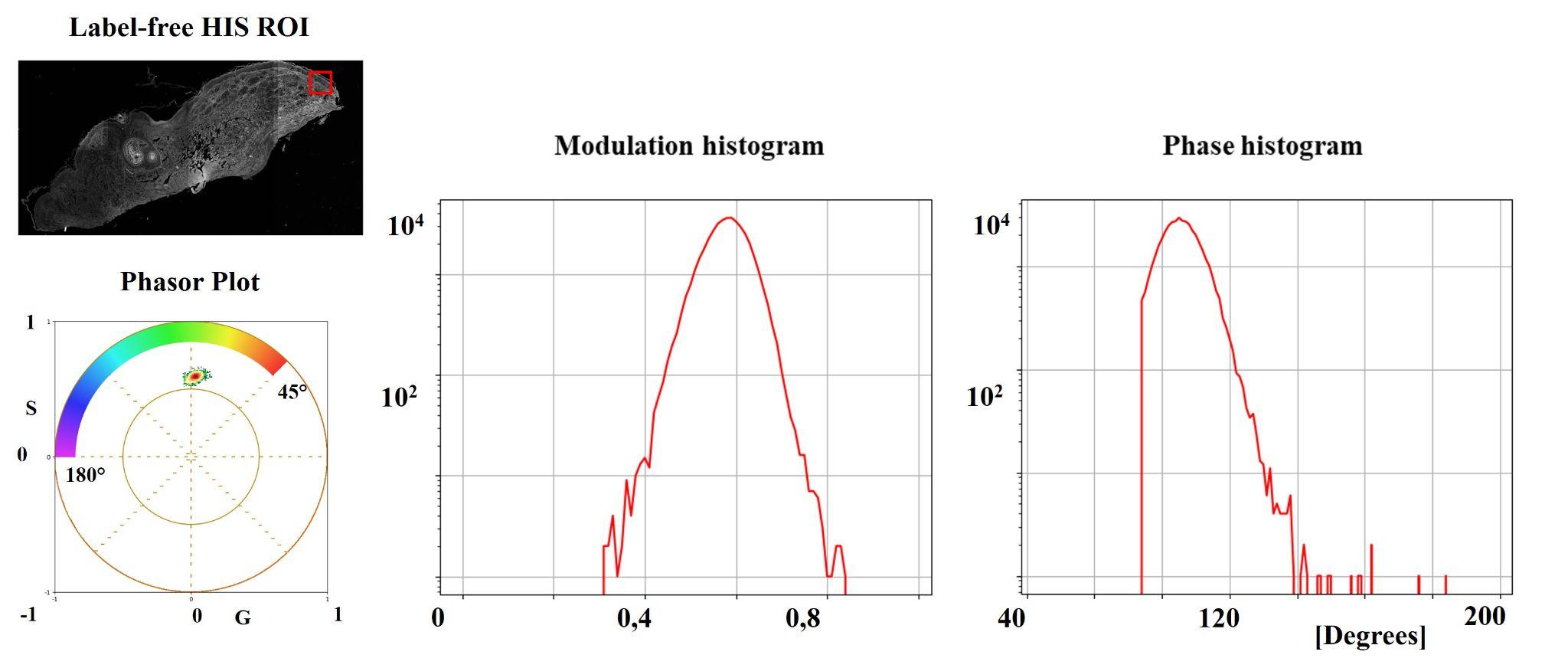


**Figure 13: Analysis of Phase and Modulation profile for ROI in “normal” tissue outside the lesion.** ROI for IN2 with non-melanocytic lesion, its associated phasor plot, and its modulation and phase histograms.

##
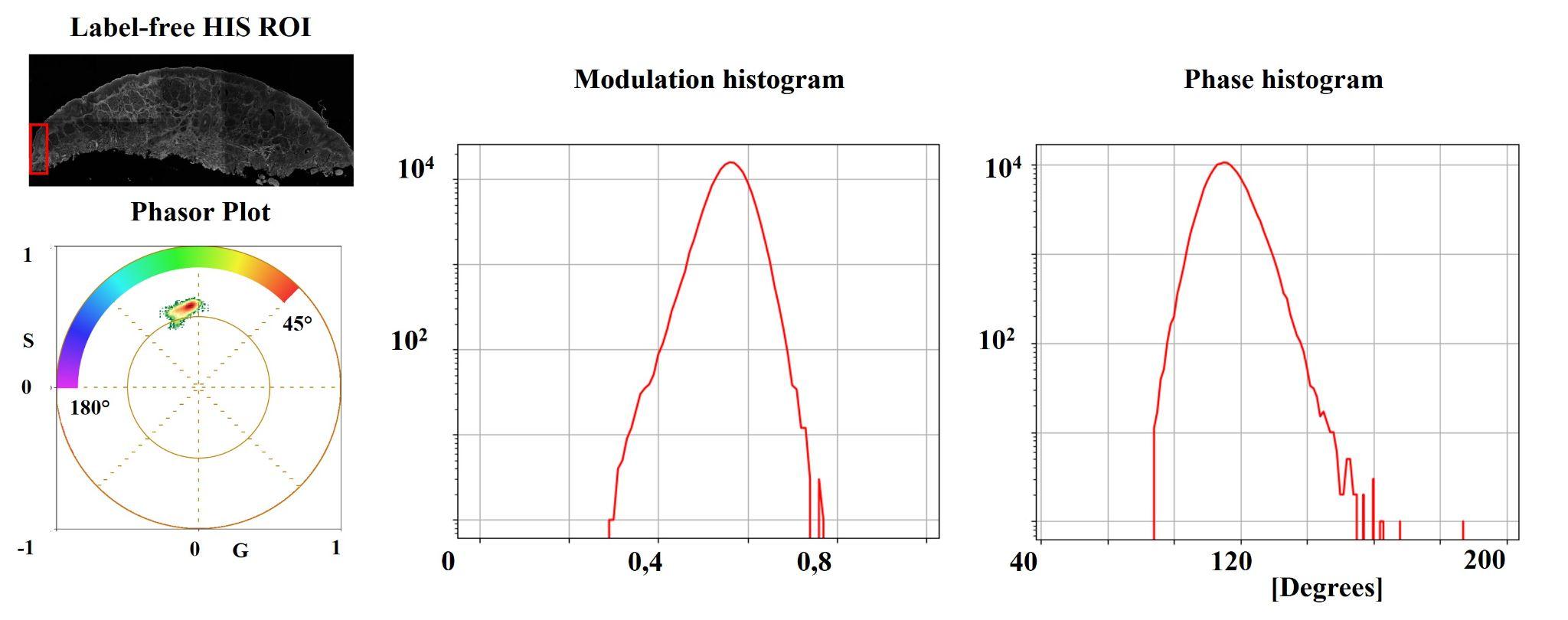


**Figure 14: Analysis of Phase and Modulation profile for ROI in “normal” tissue outside the lesion.** ROI for IN4 with non-melanocytic lesion, its associated phasor plot, and its modulation and phase histograms. Region of the IN4 with no melanocytic lesion, with its corresponding phasor plot and modulation and phase histograms.


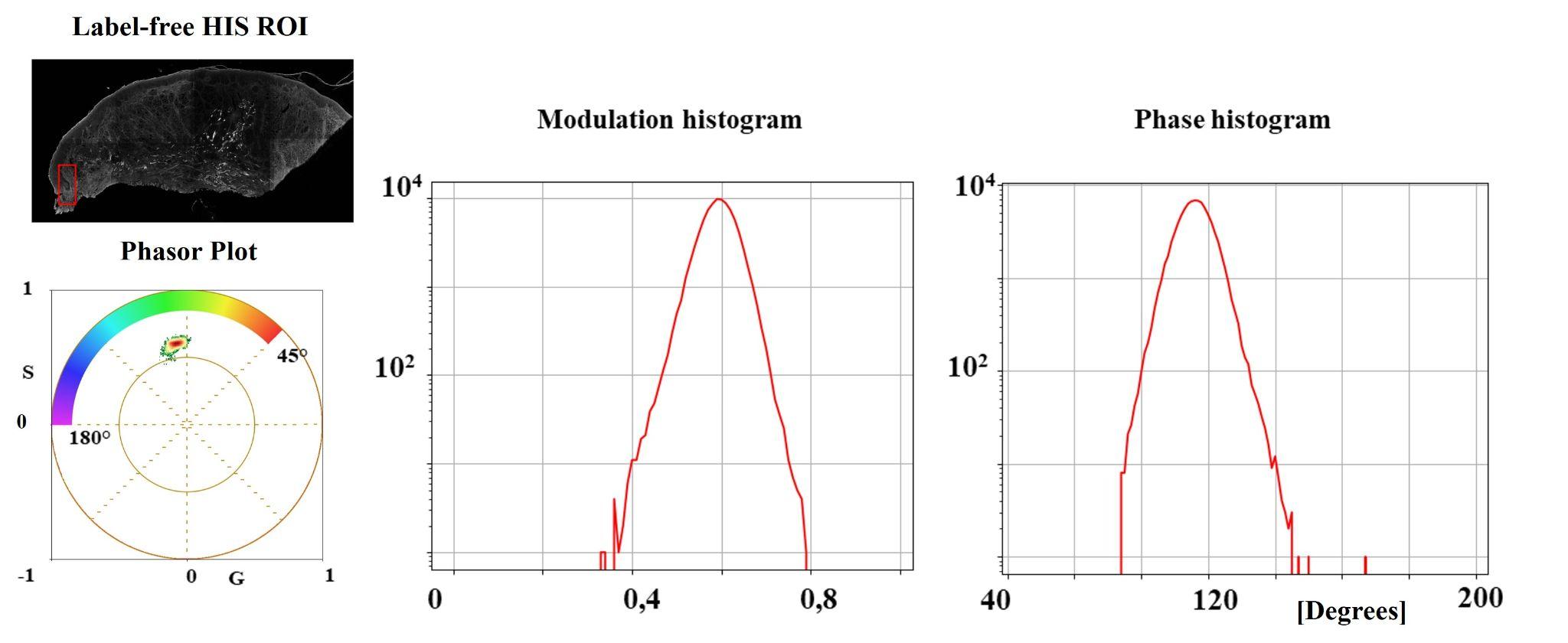


**Figure 15: Analysis of Phase and Modulation profile for ROI in “normal” tissue outside the lesion.** ROI for IN5 with non-melanocytic lesion, its associated phasor plot, and its modulation and phase histograms.
